# Robustness in population-structure and demographic-inference results derived from the *Aedes aegypti* genotyping chip and whole-genome sequencing data

**DOI:** 10.1093/g3journal/jkae082

**Published:** 2024-04-16

**Authors:** Andrés Gómez-Palacio, Gen Morinaga, Paul E Turner, Maria Victoria Micieli, Mohammed-Ahmed B Elnour, Bashir Salim, Sinnathamby Noble Surendran, Ranjan Ramasamy, Jeffrey R Powell, John Soghigian, Andrea Gloria-Soria

**Affiliations:** Department of Entomology, Center for Vector Biology & Zoonotic Diseases, The Connecticut Agricultural Experiment Station, 123 Huntington St., New Haven, CT 06511, USA; Laboratorio de Investigación en Genética Evolutiva, Universidad Pedagógica y Tecnológica de Colombia, Avenida Central del Norte 39-115, Boyacá 150003, Colombia; Faculty of Veterinary Medicine, University of Calgary, 2500 University Drive NW., Calgary, AB 2TN 1N4, Canada; Department of Ecology and Evolutionary Biology, Yale University, 165 Prospect St., New Haven, CT 06511, USA; Quantitative Biology Institute, Yale University, 260 Whitney Ave., New Haven, CT 06511, USA; Centro de Estudios Parasitológicos y de Vectores (CEPAVE), CONICET, Universidad Nacional de la Plata, Boulevard 120 s/n between Av. 60 and Calle 64, La Plata 1900, Argentina; Department of Parasitology and Medical Entomology, Tropical Medicine Research Institute, National Center for Research, Khartoum 11111, Sudan; Faculty of Veterinary Medicine, Department of Parasitology, University of Khartoum, Khartoum North 11111, Sudan; Camel Research Center, King Faisal University, P.O. Box. 400, Al-Ahsa 31982, Saudi Arabia; Department of Zoology, University of Jaffna, Jaffna 40000, Sri Lanka; Department of Zoology, University of Jaffna, Jaffna 40000, Sri Lanka; Department of Ecology and Evolutionary Biology, Yale University, 165 Prospect St., New Haven, CT 06511, USA; Faculty of Veterinary Medicine, University of Calgary, 2500 University Drive NW., Calgary, AB 2TN 1N4, Canada; Department of Entomology, Center for Vector Biology & Zoonotic Diseases, The Connecticut Agricultural Experiment Station, 123 Huntington St., New Haven, CT 06511, USA; Department of Ecology and Evolutionary Biology, Yale University, 165 Prospect St., New Haven, CT 06511, USA

**Keywords:** genotyping array, SNP chip, WGS, ascertainment bias, *Aedes aegypti*, yellow fever mosquito, demographic analyses, population structure

## Abstract

The mosquito *Aedes aegypti* is the primary vector of many human arboviruses such as dengue, yellow fever, chikungunya, and Zika, which affect millions of people worldwide. Population genetic studies on this mosquito have been important in understanding its invasion pathways and success as a vector of human disease. The Axiom aegypti1 SNP chip was developed from a sample of geographically diverse *A. aegypti* populations to facilitate genomic studies on this species. We evaluate the utility of the Axiom aegypti1 SNP chip for population genetics and compare it with a low-depth shotgun sequencing approach using mosquitoes from the native (Africa) and invasive ranges (outside Africa). These analyses indicate that results from the SNP chip are highly reproducible and have a higher sensitivity to capture alternative alleles than a low-coverage whole-genome sequencing approach. Although the SNP chip suffers from ascertainment bias, results from population structure, ancestry, demographic, and phylogenetic analyses using the SNP chip were congruent with those derived from low-coverage whole-genome sequencing, and consistent with previous reports on Africa and outside Africa populations using microsatellites. More importantly, we identified a subset of SNPs that can be reliably used to generate merged databases, opening the door to combined analyses. We conclude that the Axiom aegypti1 SNP chip is a convenient, more accurate, low-cost alternative to low-depth whole-genome sequencing for population genetic studies of *A. aegypti* that do not rely on full allelic frequency spectra. Whole-genome sequencing and SNP chip data can be easily merged, extending the usefulness of both approaches.

## Introduction

The power of genetic data to accurately infer population genetics patterns and processes can be enhanced by increasing the number of alleles at each locus and the number of loci studied. Microsatellites are highly polymorphic markers that satisfy the first criterion. They are tandem repeats abundant in most of organisms, have a high mutation rate, and are evenly distributed throughout the genome (Tautz 1989; Jonika *et al*. 2020). These attributes are desirable when assessing genetic differentiation, estimating migration, panmixia, investigating historical demographic changes, and detecting signatures of selection (Paetkau *et al*. 1995; Valdiani *et al*. 2013). Despite the widespread use of microsatellites for population genetic studies, some criticisms have been raised regarding the relatively limited number of loci (<20) commonly used for most arthropods such as mosquitoes. The accuracy in parameter estimation in most population genetic studies is more dependent on the number of markers used, rather than sample sizes (Hale *et al*. 2012). The current iteration of next-generation sequencing technologies now allows for the collection of large-scale genomic data (Davey *et al*. 2011), and population genetic studies are shifting from using a dozen or more highly polymorphic loci (often microsatellites) to the use of thousands or even millions of single nucleotide polymorphisms (SNPs) drawn from genome or transcriptome sequencing (Davey *et al*. 2011). Consequently, our ability to resolve population divergences, patterns of gene flow, perform demographic analyses, and identify signatures of selection has improved. However, despite the decreasing costs of whole-genome sequencing (WGS), and its relative convenience for some species, for species with large genomes or highly repetitive content (such as the *Aedes aegypti* mosquito; Matthews *et al*. 2018), WGS can be cumbersome and remains expensive, particularly when the studies require large numbers of individuals genotyped.

Alternatives to WGS, such as reduced-representation approaches, are increasingly common. These approaches lower costs by sequencing a consistent subset of the target genome. Perhaps most common among these techniques are genotyping-by-sequencing methods (Davey *et al*. 2011; Peterson *et al*. 2012; Ali *et al*. 2016). However, these methods suffer from marker attrition—that is, the more individuals sequenced, the fewer loci become available for robust analyses due to genotyping errors, or missing data due to low coverage (Lowry *et al*. 2017; Storfer *et al*. 2018). Another reduced-representation method involves the use high-throughput genotyping arrays (i.e. SNP chips), which are convenient for genome-wide association studies and certain population genomic studies, but may suffer from ascertainment bias (Nielsen 2004; Albrechtsen *et al*. 2010). Ascertainment bias can influence genotype frequency estimation and alter population diversity measures (Nielsen 2004; Albrechtsen *et al*. 2010). Although ascertainment bias is not a critical issue when inferring population structure (Lachance and Tishkoff 2013), a low-depth WGS approach (<10× mean coverage) is generally considered better for genome-wide genotyping in studies that require a large sample size (e.g. GWAS; Gilly *et al*. 2019).

*Aedes aegypti* is the primary vector of human arboviruses such as dengue, yellow fever, chikungunya, and Zika, affecting millions of people worldwide. Population genetic studies in this species are essential to inform local vector surveillance and control programs worldwide about the demographic (i.e. population expansion, contraction, migration rates, and dispersal ranges) and genetic attributes (i.e. insecticide resistance genes) of their populations (Kotsakiozi, Evans, *et al*. 2018; Endersby-Harshman *et al*. 2020). Genetics-based studies have also provided valuable information about the evolutionary history of *A. aegypti* and its global invasion pathways, and are key to understanding the impact of human activity in the dispersal of this important disease vector at different spatial-temporal scales (Powell *et al*. 1980, 2018; Rose *et al*. 2023).

Research of the population genetics of *A. aegypti* has historically relied on allozymes and microsatellite markers (e.g. Powell *et al*. 1980, Gloria-Soria *et al*. 2016). More recently, there has been a shift to SNP discovery based methods such as WGS (Lee *et al*. 2019), RAD-seq (Brown *et al*. 2014; Rašić *et al*. 2016), exome-capture based sequencing (Crawford *et al*. 2017), and SNP chip genotyping (Evans *et al*. 2015; Kotsakiozi, Evans, *et al*. 2018; Kotsakiozi, Gloria-Soria, *et al*. 2018; Evans *et al*. 2019; Soghigian *et al*. 2020). However, concerns have been raised regarding the compatibility of the data generated by the different research groups and platforms (Schmidt *et al*. 2021), which may restrict the extent to which these genetic databases could be combined.

The Axiom aegypti1 chip (Evans *et al*. 2015; hereafter, SNP chip) is a high-throughput SNP genotyping array designed to provide genetic information across the entire genome of *A. aegypti* while overcoming the challenges imposed by a large genome size and the prevalence of highly repetitive regions and transposable elements. The SNP chip contains a total of 50,000 SNPs, from which ∼25,000 have been validated and can effectively differentiate populations around the world (Evans *et al*. 2015; Kotsakiozi, Evans, *et al*. 2018; Kotsakiozi, Gloria-Soria, *et al*. 2018; Evans *et al*. 2019; Soghigian *et al*. 2020). The *A. aegypti* SNP chip has also been used to perform genome-wide association studies (Dickson *et al*. 2018; Cosme *et al*. 2022). It is generally assumed that the results from SNP chip analyses are similar/compatible with results obtained by other methods, such as whole-genome sequence data (WGS) and reduced-representation genomic libraries (e.g. ddRADs), although this has not yet been evaluated.

Here, we compare different population genetic metrics derived from the *A. aegypti* SNP chip and low-coverage WGS to determine the consistency and compatibility among these datasets and examine the advantages and disadvantages of using each genotyping method for population genetic studies.

## Methods

### *Aedes aegypti* collections and genotyping

We used a total of 92 individual *A. aegypti* mosquitoes for different analyses based on previously published works (Evans *et al*. 2015, 2019, Kotsakiozi, Evans, *et al*. 2018, Kotsakiozi, Gloria-Soria, *et al*. 2018, Soghigian *et al*. 2020, Elnour *et al.* 2022) to represent the *A. aegypti* distribution inside and outside Africa (Supplementary Tables 1 and 2). Sixty-two mosquitoes were genotyped at 50,000 loci using the Axiom aegypti1 SNP chip (Life Technologies Corporation CAT#550481; Evans *et al*. 2015) at the University of North Carolina Functional Genomics Core, Chapel Hill. The genome of 30 individuals were sequenced at the Marine Biological Laboratory in Woods Hole MA from up to 100 ng of DNA using an insert size of approximately 400 bp, with the sequencing libraries prepared individually using the Nugen Ultralow V2 protocol (#0344) with 7–10 cycles of library amplification. Equimolar quantities of each library were pooled and size selected to 570 bp using a Pippin Prep (Sage Science) and sequenced on an Illumina NextSeq 500 with the paired end high throughput protocol (2 × 150) for a target coverage of 1.5×. Additionally, we sequenced 13 individuals (target coverage: 10×) that had been genotyped previously with the Axiom aegypti1 SNP chip at the Yale Center for Genome analysis using an insert size of approximately 200 bp and the NEBNext Ultra II DNA Library Prep Kit for Illumina #E7645 with 10 PCR cycles and the NovaSeq S4 2 × 150 platform.

### SNP chip reproducibility

We processed 20 individuals from Sudan and Sri Lanka in triplicate to evaluate the consistency of the SNP chip genotypes. We filtered all 50,000 SNPs generated by the chip in PLINK v. 1.9 (Purcell *et al*. 2007) and allowed for a maximum missing data per marker of 0.2, a per individual missing data of 0.05, and minor allele frequency < 0.01 (--geno 0.2 –mind 0.05 maf 0.01). We then compared the replicates from each individual using the GenotypeConcordance tool from Picard v. 2.9.0 (Broad Institute 2019).

### Annotating genomic positions of SNPs

Since the original coordinates for the SNP chip were based on an earlier version of the *A. aegypti* reference (Evans *et al*. 2015), we confirmed and updated the coordinates of all SNPs prior to performing concordance analyses by annotating the genomic positions of the SNP chip probes on the AaegL5 reference genome (Matthews *et al*. 2018). To do this, we first created a local blast database from the AaegL5 reference genome using makeblastdb and mapped the position of all 50,000 probes using blastn, both part of the NCBI blast suite v. 2.12.0+ (Sayers *et al*. 2022). We then identified probes that mapped uniquely to the reference genome and kept only those for further analyses. The updated SNP chip coordinates are provided in BED format as Supplementary File 3.

### WGS genome mapping and genome-wide SNP calling

We filtered the paired-end short-reads obtained from Illumina sequencing (WGS) by read quality (q < 30) and removed adapters using fastp v. 020.1 (Chen *et al*. 2018), with subsequent removal of PCR duplicates and grouping of reads using Picard v. 2.9.0 (https://broadinstitute.github.io/picard/). We then mapped reads from each mosquito to the AaegL5 reference genome using bowtie2 v. 2.4.5 (Langmead and Salzberg 2012: 2), and sorted and indexed these outputs using SAMtools v. 1.15 (Danecek *et al*. 2021). We then called variants at positions that corresponded to the SNP chip loci with probes that mapped uniquely to the reference genome using the *mpileup* function, then removed indels using the *call* function, both from BCFtools v. 1.16 (Danecek *et al*. 2021). We filtered out non-biallelic sites in PLINK v. 1.9 (Purcell *et al*. 2007). From these VCF outputs, we retained raw sequencing depth (DP) and Phred quality score (QUAL) to assess genotype concordance between SNP chip and WGS statistically (see below).

### Genotype concordance between WGS and SNP chip

We used vcfR v. 1.13.0 (Knaus and Grünwald 2017) to import VCF files into R v. 4.2.1 (R Core Team 2021) and counted the number of concordant, discordant, and missing sites for each individual. We then calculated mean genotype concordance for each individual as the number of concordant sites divided by the sum of concordant and discordant sites. We also calculated allele sensitivity for the WGS and SNP chip. We define allele sensitivity (or variant sensitivity percentage) as the number of concordant sites between WGS and SNP chip containing the alternative allele divided by the total number of SNPs with the alternative allele in either the SNP chip or WGS. To assess how sequencing depth and quality affected the log-odds of concordance between WGS and the SNP chip, we used 2 generalized linear mixed models (GLMM) with a binomial (logit) link function. We mean-centered and scaled sequencing depth and quality score (both *n* = 410,746) to unity and included each as a fixed effect and individual (*n* = 12) as a random effect and fitted both models using the *glmer* function in lme4 v. 1.1–30 (Bates *et al*. 2015). We do not consider a model with both fixed effects together because sequencing depth is required to calculate quality score, and thus is collinear (Supplementary Fig. 1). To estimate 95% confidence bands, we used the *predictInterval* function in merTools v. 0.5.2 (Knowles *et al*. 2020), which creates a sampling distribution based on the model parameters and estimates fitted values across that distribution. This is a faster alternative to bootstrapping. We used the *r.squaredGLMM* function in MuMIn v. 1.47.1 (Bartoń 2018) to estimate marginal (*R*^2^_M_) and conditional (*R*^2^_C_) coefficients of determination to assess model fit. These statistics are similar to the coefficient of determination (*R*^2^), but respectively account for variance explained by the fixed effects alone and the total variance explained by fixed and random effects (Nakagawa and Schielzeth 2013; Nakagawa *et al*. 2017). Lastly, using these concordance data, we separately tested whether concordance between SNP chip and WGS differed between the global invasive and African native populations using a 1-way ANOVA.

### SNP subsampling and SNP chip bias

We evaluated the effect of the number of SNPs analyzed on estimates of genetic diversity using individuals that were genotyped with both methods. We compared subsamples containing similar number of SNPs randomly drawn from each dataset using the SelectVariants option in GATK v. 4.1.2.0 (DePristo *et al*. 2011; Van der Auwera and O’Connor 2020). We generated 3 subsamples containing 46,962; 24,127; and 4,016 SNPs from the WGS dataset and 2 subsamples containing 23,331 and 4,710 SNPs from the SNP chip. The first WGS subset was meant as a direct comparison to the complete SNP chip dataset. The second and third subsets represent roughly 50 and 10% of the loci captured by the SNP chip dataset. We then calculated individual mean observed heterozygosity (Het) using the *stats* option from BCFtools v. 1.9 (Danecek *et al*. 2021) for the complete dataset (i.e. 46,962 for SNP chip and 208,775 for WGS) and each subset and tested whether the distributions differed between sequencing platforms among equivalent subsamples and across them, using a Kruskal–Wallis test followed by Dunn's tests post hoc to understand specific differences. We also calculated the folded Site Frequency Spectra (SFS) for both datasets using the software dadi (Gutenkunst *et al*. 2009) and compared it with the true SFS using maximum-likelihood numerical optimization based on simulations of 50,000,000 sites drawn from the WGS data using the *realSFS* option implemented in ANGSD v 0.933 (Nielsen *et al*. 2012; Korneliussen *et al*. 2014).

### Genetic diversity, structure, and demographic history

We investigated the effect of the SNP chip ascertainment bias on estimates of population diversity by comparing groups of 5 individuals from 6 populations (*N* = 30 individuals; Supplementary Table 2). Although each population was represented in both the SNP chip and WGS dataset, most individuals did not overlap between the methods. We filtered both datasets based on minor allele frequency, maximum missing genotype rate, and linkage using -maf 0.1 -geno 0.1 -indep-pairwise 50 10 0.3 parameters, respectively, in PLINK 1.9 (Purcell *et al*. 2007).

We performed principal component analysis (PCA) and Bayesian clustering analysis using the *pca* and *snmf* functions in the LEA v. 3.3.2 package (Frichot and François 2015) for R. We tested K = 2–6 ancestral populations for Bayesian analysis using the entropy method with 1,000,000 iterations with 10 repetitions. We imputed missing genotypes as necessary by using ancestry and genotype frequency estimates with the *impute* function in the same software. We selected the best *K-*run using the *which.min* function for cross-entropy, also from the LEA R package (Frichot and François 2015).

We calculated the Weir and Cockerham weighted *F*_ST_ (Weir and Cockerham 1984) from 2 separate datasets: (1) within groups and (2) between groups for both methods, and compared African and non-African groups as well as pairwise population values using VCFtools v 0.1.14 (Danecek *et al*. 2021). We used a least-squares regression to test whether pairwise *F*_ST_ between SNP chip and WGS were related.

We used approximate Bayesian computation methods (ABC; Beaumont *et al*. 2002) as implemented in DIYABC-RF v.2.1 (Collin *et al*. 2021) and tested 4 scenarios of migration among continents using the SNP chip and WGS datasets, independently. After the input databases were filtered by the program using default parameters (no monomorphic loci and minor allele frequency [MAF] < 5%), the SNP chip dataset contained 17,353 SNPs and the WGS dataset 178,004 SNPs. Subsequently, we performed simulations from a similar number of random loci drawn from each platform (17,353 SNPs for SNP chip and 17,400 SNPs for WGS). The scenarios and parameter priors were identical to those used by Gloria-Soria *et al*. (2016) for microsatellites (Supplementary Table 3). In brief, these scenarios are: (1) Africa to America to Asia; (2) Africa to Asia to America; (3) Africa to America + Africa to Asia (after colonization of the Americas); and (4) Africa to America + Africa to Asia (before colonization of the Americas). We assessed the quality of the predictions using the global prior error (across all scenarios) as well as local posterior error (i.e. 1—posterior probability; Chapuis *et al*. 2020) using Random Forest as implemented in DIYABC-RF with 10 replicates for each dataset.

We evaluated the evolutionary relationships among populations of *A. aegypti* using maximum-likelihood inference with both SNP chip and WGS datasets, and the program IQ-TREE v2.1.3 (Minh *et al*. 2020: 2). We estimated branch support with the ultrafast bootstrap (Hoang *et al*. 2018) and selected the best-fitting substitution model using ModelFinder (Kalyaanamoorthy *et al*. 2017), both implemented in IQ-Tree.

### Merging genotypes from the SNP chip and WGS

We evaluated the compatibility of WGS and SNP chip data in the same analyses by merging previously published SNP data and WGS data from 5 populations. Individuals did not overlap between the WGS and SNP chip datasets and in some instances originated from different temporal collections (Supplementary Tables 1 and 2). We used *Aedes mascarensis* WGS data as outgroups. We mapped WGS data as described in the *WGS genome mapping and genome-wide SNP calling* methods section. We annotated the VCF file containing WGS loci with SNP chip position information, then converted the VCF file to PLINK's bed format, and removed SNP locations not found on chromosomes. Next, we retrieved previously published SNP data in a PLINK bed file from the same populations for which we had WGS data. We then followed the PLINK manual for merging PLINK bed files, which begins by correcting SNPs with flipped orientation and identifying SNPs that cannot merge due to the presence of a third allele. A very small number of SNPs in WGS data had a third allele (see *Results*, below) and were excluded during merging. Following merger, we considered only SNPs found to be concordant across at least 50% of samples (see *Genotype concordance between WGS and SNP chip* section above). We then followed our standard protocol for analyzing population genetic data as if it were derived from the Axiom Array (Gloria-Soria *et al*. 2018, 2022; Soghigian *et al*. 2020): we included only SNPs that had been previously shown to exhibit Mendelian inheritance patterns, excluded any SNP with more than 20% missing data, and removed linked SNPs using the PLINK command –indep 75 kb 50 2. We provide the scripts used to merge WGS and SNP chip data as part of supplementary data (Supplementary File 1).

Following the merge, we performed a series of typical population genetic analyses to evaluate the compatibility of WGS and SNP chip data. We calculated *F*_ST_ between populations and dataset types using stamppFst from the R package StAMPP v1.6.3 (Pembleton *et al*. 2013). We then performed sparse nonnegative matrix factorization analysis (Frichot and François 2015), a fast and accurate STRUCTURE-like method, with the function snmf from the R package LEA v3.6.0 (Frichot and François 2015; Gain and François 2021). We followed authors' recommendations to choose the best K, by examining the lowest cross-entropy across runs, but we also visualized K = 7 as this was the number of populations in our analyses. We then performed a PCA in R using the function dudi.pca from the package ade4 v1.7–19 (Dray and Dufour 2007), and a phylogenetic analysis in IQTree 2 (Minh *et al*. 2020) with 1000 Ultrafast Bootstrap replicates under the GTR + F substitution model, similar to above. Finally, we excluded *A. mascarensis* samples, and evaluated how well SNP chip data alone could assign the WGS data (subset to the SNP chip positions, as described above) back to their populations of origin with the R functions dapc and predict.dapc from the package adegenet v2.1.7 (Jombart 2008). We followed author’s tutorials and used 11 as the number of PCA axis and 5 as the number of discriminant functions, accordingly, based on examination of cross validation plots.

## Results

### SNP chip reproducibility

After quality filtering, 38,306 SNPs remained of the 50,000 SNPs genotyped. We found mean genotype reproducibility to be 36,007 ± 1,915 (94 ± 5%; mean ± SD) when comparing genotypes produced by 3 independent SNP chip runs for 20 distinct individuals, ranging from 91 ± 6% to 96 ± 3% (Supplementary Table 4). A 2-sample *t*-test revealed no statistical difference in genotype reproducibility based on source population [*t*(15.601) = 0.165, *P* = 0.871].

### Genotype concordance between SNP chip and WGS

A total of 43,593 SNPs out of 50,000 SNPs on the Axiom aegypti1 SNP chip were uniquely mapped to the 3 chromosomes on the *A. aegypti* AaegL5 reference genome (Matthews *et al*. 2018), while another 265 mapped uniquely to unplaced contigs. We analyzed only those SNP positions that mapped uniquely to chromosomes. Our 12 samples averaged a sequencing depth of 6.37 ± 0.77 (mean ± standard deviation) and a quality score of 117.36 ± 8.62 (Supplementary Table 6) for the positions we extracted. We detected 34,228.83 ± 1,546.11 (78.52 ± 3.55%) (mean ± standard deviation) common SNPs on both WGS and SNP chip, with samples from Europa Island (30,992; 76.09%) and Mexico (36,689; 84.16%) representing the minimum and maximum, respectively (Supplementary Table 6). Of those present in both platforms, 28,325.58 ± 2,537.94 (82.72 ± 5.91%) had concordant genotypes, with samples from Georgia (22,485; 67.79%) and Mexico (31,668; 86.31%) representing the minimum and maximum numbers of concordant genotypes, respectively (Supplementary Table 5). We found 26,353.5 ± 1,971.82 (93.18 ± 2.28%) concordant homozygous sites, with Argentina (28,090; 90.3%) and Georgia (22,003; 97.86%) representing minimum and maximum numbers of concordant homozygous sites, respectively (Supplementary Table 5). Across all samples, we found greater sensitivity to heterozygotes in the SNP chip (mean ± SD = 76.94 ± 19.39%) than low-depth WGS (mean ± SD = 31.73 ± 9.93%) (Supplementary Table 5). A 1-way ANOVA showed no differences in concordance between the SNP chip and WGS across mosquitoes sampled in Africa and outside of Africa (*F*[1,10] = 0.042, *P* = 0.843). Lastly, we identified 20,841 SNPs that were consistently concordant in at least 90% of the samples when present, 31,685 SNPs that were consistently concordant across at least 75% of the samples when present, and 40,047 SNPs that were consistently concordant across at least 50% of the samples when present. We provide probe IDs of these SNPs and those that mapped uniquely to the AaegL5 reference genome chromosomes in Supplementary File 2.

**Fig. 1.**
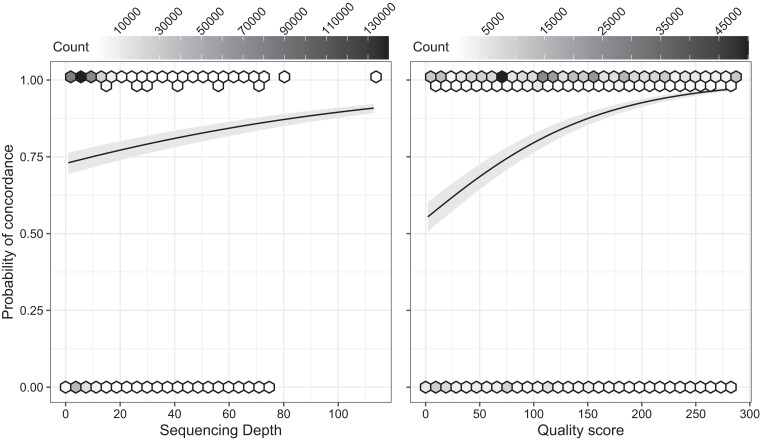
Probability of concordance between WGS and SNP chip modeled using sequencing depth and quality score. Each detected SNP across 12 WGS sampled individuals is binned into a hexagon. Darker shades indicate higher density of SNPs in a bin. Lines show the generalized linear mixed model (with logit link function) slope after converting log-odds to probabilities. Gray band are 95% confidence bands.

Both GLMM fitted showed increased log-odds of concordance between WGS and the SNP chip as sequencing depth and quality score of the WGS increased. Specifically, unit increases in sequencing depth increased the odds of concordance by a factor of nearly 1.5, while unit increases in quality score increased the odds of concordance by a factor of nearly 2.6. In terms of probability, even at low sequencing depths and quality scores (i.e. <5 and <10, respectively), we found moderately high rates of concordance (Fig. 1). While slope estimates (and thus, odds ratios) for both models were significant (*P* < 0. 001), quality score better modeled the probability of concordance between WGS and SNP chip genotypes (Supplementary Table 6).

### Effect of ascertainment bias on genetic diversity estimates

**Fig. 2.**
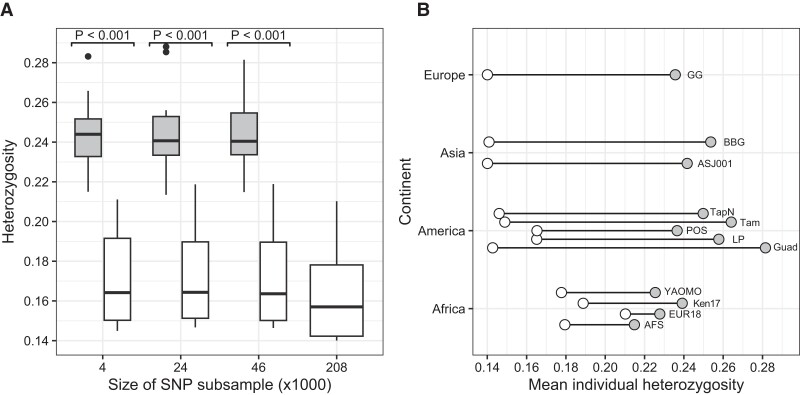
Differences in heterozygosity between SNP chip (gray boxes and circles) and whole-genome sequencing (WGS; white boxes and circles). a) Boxplots of heterozygosity detected for each platform at each subsample (*n* = 12 specimens for each subsample). Bold black bars show medians, while boxes show inter-quartile range. Lines that extend from boxes show 95% confidence intervals, and black dots found beyond the lines are outlier observations. b) Dumbbell plot showing mean individual heterozygosity of WGS (208,000 SNPs) and Axiom Aegypti1 SNP chip (46,000 SNPs) for 12 sampling localities grouped by continent. GG, Georgia; BBG, Cebu City, Philippines; ASJ001, Jeddah, Saudi Arabia; TapN, Tapachula, Mexico; Tam, Tampa, USA; POS, Posadas, Argentina; LP, La Plata, Argentina; Guad, Guadeloupe Island, France; YAOMO, Yaounde, Cameroon; Ken17, Kaya Bomu, Kenya; EUR18, Europa Island, France; AFS, Johannesburg, South Africa.

For the 12 individual samples sequenced in parallel with both SNP chip and WGS, we recovered an average of 208,775 ± 6 biallelic SNPs from the WGS data, with average coverage of 30.4× and a minimum coverage of 23.9× (Guadeloupe Island; Supplementary Table 7). When subsampled by SNP number (Supplementary Table 3), we found significant differences between platforms (Kruskal–Wallis test χ^2^ (6) = 61.011, *P* < 0.001). Post hoc Dunn's test revealed that the SNP chip detected greater heterozygosity than WGS across each subsample (*Z* = −4.03 to −3.96, all *P* < 0.001; Fig. 2a). Even at the individual level, we found higher mean individual Het values for SNP chip than for WGS for African samples (Fig. 2b, Supplementary Table 8). We also found that allele frequency spectrum differed between “corrected” (i.e. “true” spectrum), WGS, and SNP chip (Supplementary Fig. 2). The SFS showed a clear bias toward intermediate frequency alleles in the SNP chip and those of the WGS showed higher number of SNPs toward low frequencies (Supplementary Fig. 2).

### Concordance in population differentiation between SNP chip and WGS

Quality filtering the SNP chip data yielded 17,358 SNPs, while we obtained 178,004 SNPs from WGS data (*N* = 30 for both datasets). PCA showed minor differences between the datasets, with both providing sufficient resolution to separate African and outside of Africa populations (Supplementary Fig. 3). Furthermore, both datasets placed Argentina (El Dorado population) as an intermediate group (Supplementary Fig. 3).

Bayesian clustering analysis identified K = 2 as the optimal number of genetic clusters in both datasets, according to minimum cross-entropy value (Supplementary Fig. 4). We found no significant differences in the attributed genetic ancestry of populations from Africa and outside of Africa among the datasets (paired *t*-test: *t*(29) = −0.682, *P* = 0.500). For both datasets, individuals from Argentina (El Dorado) showed clear mixed ancestry from Africa and outside Africa genetic clusters.

**Fig. 3.**
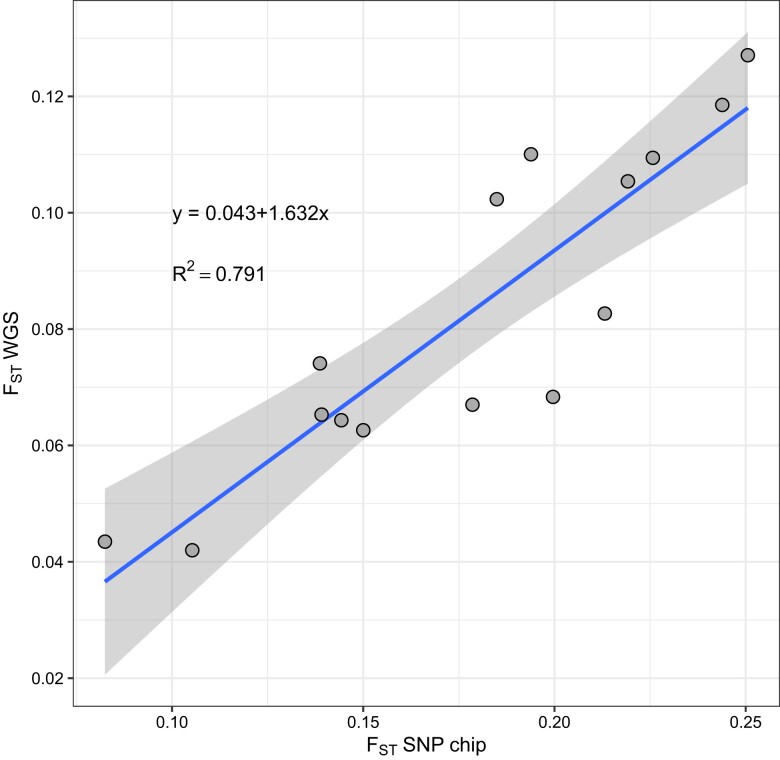
Scatterplot showing the relationship between Weir and Cockerham *F*_ST_ measured for SNP chip and WGS from 6 populations. Each dot shows *F*_ST_ for each pair of populations. The line shows the ordinary least-squares regression slope and the shaded band shows the 95% confidence interval of the slope.

The distribution of per site (*F*_ST_) genetic differentiation within groups was similar between WGS and SNP chip (Kruskal–Wallis *H-*test; χ^2^(1,335) = 328, *P* = 0.549; Supplementary Fig. 5). Estimates of population genetic differentiation among Africa and outside Africa were *F*_ST_ = 0.12 and 0.06 for the SNP chip and WGS, respectively. Ordinary least-squares regression between *F*_ST_ of SNP chip and WGS showed a significant, positive relationship (*R*^2^ = 0.791, *F*(1,13) = 49.13, *P* < 0.001; Fig. 3; Supplementary Table 9), indicating that any interpretation of genetic differentiation derived from either WGS or SNP chip will be congruent with the other, but may vary in magnitude.

**Fig. 4.**
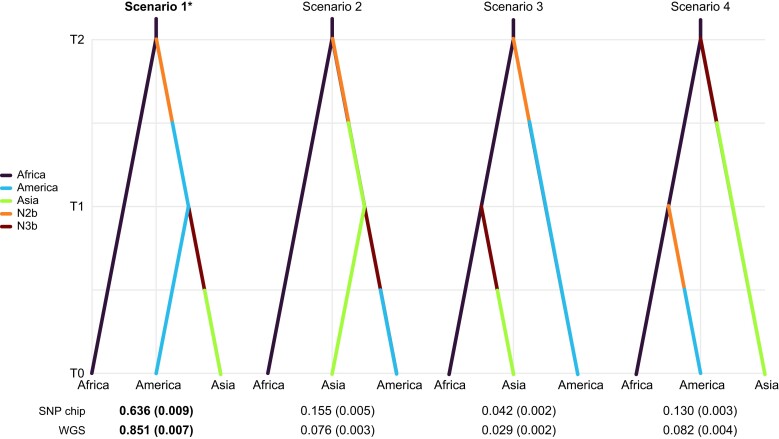
Evolutionary scenarios of *A. aegypti* at continental-scale, evaluated using SNP chip and WGS datasets. T0 represents the most recent time point and increasing values of T go back in time. Scenario 1: Africa to America to Asia; scenario 2: Africa to Asia to America; scenario 3: Africa to America + Africa to Asia (after America colonization); and scenario 4: Africa to America + Africa to Asia (before America colonization). Each region is presented with different colors, as shown in the respective inset. Ancient hypothetical populations under bottleneck process are denoted as N2b and N3b. Posterior probability for the best scenario after Random Forest analysis is indicated by an asterisk and in bold as obtained for each method.

Demographic analysis using approximate Bayesian computation (ABC) supported the global colonization pathway of *A. aegypti* described by scenario 1 (from Africa to America to Asia) (Supplementary Table 3; Fig. 4) with both SNP and WGS datasets (posterior probability = 0.636 ± 0.009; 0.851 ± 0.007, respectively). We estimated that American populations split from African populations 5,282 (±462) and 5,347 (±419) generations ago from the SNP chip and WGS, respectively. While ABC using SNP chip data exhibited local error rates (i.e. 1—posterior probability; Chapuis *et al*. 2020) nearly double that of WGS, both datasets had similar global priors, which indicated high accuracy and quality of the predictions (Supplementary Table 3). From these analyses, we can infer the split of Asian populations from those in America to be 1,438 (±168) and 1,526 (±144) generations ago with the SNP chip and WGS data, respectively (Supplementary Table 3). Assuming *A. aegypti*, averages 10 generations per year (Gloria-Soria *et al*. 2016), the African and American populations would have diverged approximately 500 years ago, and Asian and American populations approximately 120 years ago.

The SNP chip and WGS datasets yielded nearly identical phylogenies, depicting 2 main clades that contain African and outside of Africa populations (Supplementary Fig. 6). However, nodes with low support on tree derived from the SNP chip dataset were strongly supported in the tree generated from WGS. Both trees placed El Dorado, Argentina populations as basal to the populations outside Africa (Supplementary Fig. 6).

### Compatibility of SNP chip and WGS-derived genotypes

**Fig. 5.**
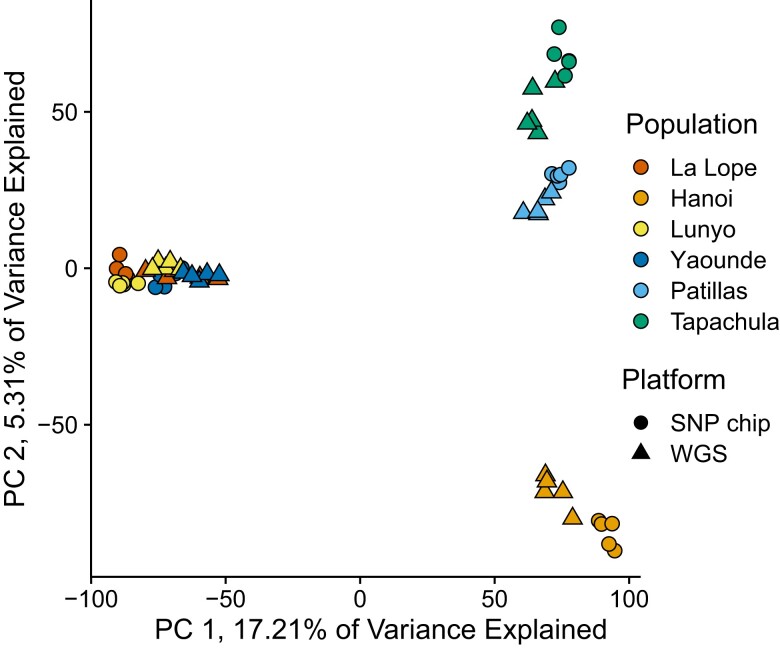
Principal component analysis of combined WGS and SNP chip data for positions validated in this manuscript. Main population clusters are driven by geographic sampling region. Each locale is drawn in a different color, as indicated by the legend. The source populations are: La Lope—La Lope Forest, Gabon; Hanoi—Hanoi, Vietnam; Lunyo—Lunyo, Uganda; Yaounde—Yaounde, Cameroon; Patillas—Patillas, Puerto Rico; Tapachula—Tapachula Norte, Mexico.

**Fig. 6.**
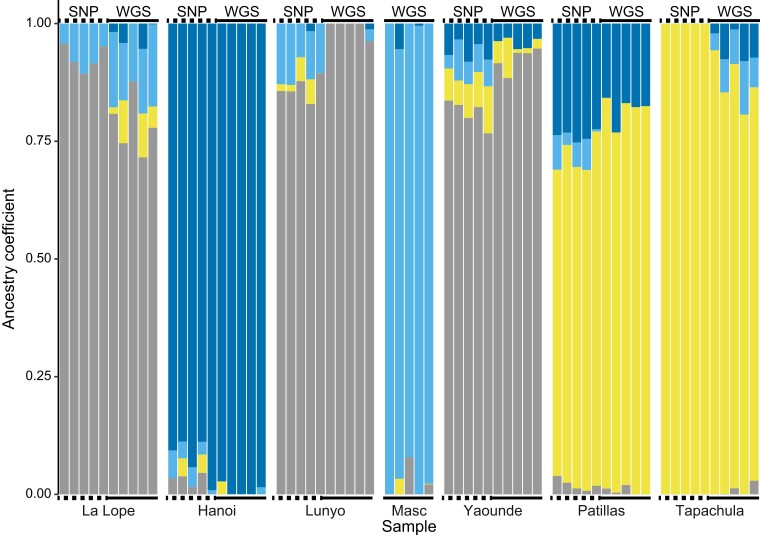
Ancestry coefficients for combined WGS and SNP chip dataset, for positions validated as concordant in this manuscript. We found that K = 4 was the optimal number of clusters based on cross-entropy. Samples cluster by geographic region, regardless of genotyping method. The source populations are: Masc—*Aedes mascarensis* outgroup (only WGS data); La Lope—La Lope Forest, Gabon; Hanoi—Hanoi, Vietnam; Lunyo—Lunyo, Uganda; Yaounde—Yaounde, Cameroon; Patillas—Patillas, Puerto Rico; Tapachula—Tapachula Norte, Mexico.

The WGS and SNP chip data were highly compatible. Only 26 positions contained tri-allelic sites in WGS data and had to be excluded from merging. Following merging and filtering, the final dataset contained 17,175 variants with a total genotyping rate of 95.27%. Our population genetic analyses resulted in qualitatively similar population structure between data acquisition methods. Across pairwise *F*_ST_ estimates, we found the lowest estimates to be between SNP chip and WGS data from the same population (Supplementary Table 10). Population differentiation was observed in the PCA of the merged dataset regardless of the genotyping platform (Fig. 5). We found K = 4 to be the optimal number of clusters based on cross-entropy, and visualization at this K places all WGS and SNP samples in the same population clusters (Fig. 6). While we found that the same populations cluster together at K = 7 as in K = 4 across 3 PC axes (Supplementary Fig. 7), we found greater uncertainty within native African forest populations (e.g. between Uganda, Cameroon, and Gabon) than outside Africa. In phylogenetic analyses, all populations resolve together, save for 3 individuals from Gabon in the WGS dataset, which resolved at the base of the clade containing all *Aedes aegypti formosus* samples (Supplementary Fig. 8). These 3 individuals had the highest amount of missing data, an average of 22%. Excluding these samples, WGS samples averaged 5.1% missing data. Accuracy of our DAPC-based population assignment test using SNP chip data as reference panel to assign WGS samples to populations was 83% successful (Supplementary Fig. 9), with all assignment errors involving Gabon.

## Discussion

The Axiom aegypti1 SNP chip yields highly reproducible genotypes and is more sensitive than a low-coverage WGS approach in detecting alternative alleles. The global population structure of *A. aegypti* detected using the SNP chip is consistent with that derived from WGS, as is the topology of the phylogenetic tree. However, genetic distance and diversity estimates in populations outside of Africa are magnified in the SNP chip analyses. This is consistent with the selection criteria used to choose SNP loci for the chip: aiming to equally represent Africa and outside Africa diversity, despite Africa having been overall more genetically variation (Evans *et al*. 2015). Regardless of the bias in SNP chip design, similar demographic histories could be inferred from the 2 datasets, with WGS data leading to higher posterior support for the scenario of choice, and lower local error rates. Thus, while results generated with the SNP chip are compatible with those from WGS, it is important to keep in mind the biases described above and correct them, when possible. Here, we have also addressed the compatibility between the SNP chip databases and WGS and demonstrated that with the appropriate quality filters, both databases can be combined and will enhance each other.

### Replicability and reproducibility of genotypes

We found the Axiom aegypti1 SNP chip to be highly replicable and reproducible. Three independently generated genotyping runs for the same individual in different chips generated 36,007 ± 1,915 (94 ± 5%; mean ± SD) concordant genotypes. We found no differences in genotype replicability between African (native range) and Asian populations (invasive range), suggesting that the observed genotyping variability among individuals and populations is likely due to differences in DNA quality of individual samples (i.e. concentration and integrity), rather than the source population.

### Concordance between WGS and SNP chip

Our analyses showed a genotype concordance of 80% between the Axiom aegypti1 SNP chip and WGS genotypes in more than half of the specimens. We found that more than 20,000 SNP sites are concordant across 90% of the specimens when a low-depth sequencing approach is used. Modeling probability of concordance based on sequencing depth and quality scores suggests that concordance is possible even at low values for both parameters, with minor increases in quality score dramatically increasing concordance probability (Fig. 1). The association between concordance, depth, and quality suggest that the low-depth resequencing approach likely underestimates “true” concordance between platforms (Fig. 1 and Supplementary Fig. 1). This is further supported by the fact that we found higher concordance among homozygous SNP positions than among heterozygous SNP positions. Given the increased depth necessary to reliably recover heterozygous positions, this highlights the ability of the Axiom aegypti1 SNP chip to detect heterozygous positions missed by low-coverage WGS, which are relevant for complex trait association studies (Cosme *et al*. 2022, Dickson *et al*. 2018).

### Ascertainment bias of the Axiom aegypti1 SNP chip

#### Allele frequency spectrum shift

We observed differences in allele frequency spectrum of genotypes generated by the Axiom aegypti1 SNP chip compared to the “true” simulated and WGS spectrum. Because SNP frequency determines the probability of recovering alleles in a population, low-frequency or rare alleles are likely to remain undiscovered, while those at intermediate frequency will be over-represented (Sethupathy and Hannenhalli 2008). The ascertainment bias we observe in the SNP chip results is due to the over-representation of alleles chosen during the SNP chip development to maximize the genetic differentiation among the populations (Evans *et al*. 2015) and likely missing population-specific alleles that may not have been represented in the original dataset. Thus, since population-specific variation generally occurs at lower frequency, population inferences using the SNP chip based on allele frequency (such as *F*_ST_) may be over- or underestimated depending on the degree of inter-population differentiation with the ascertained sample from which the SNPs were originally selected (Albrechtsen *et al*. 2010).

#### Genetic diversity

We detected greater heterozygosity from the SNP chip than from WGS regardless of the number of SNPs considered. However, mean individual heterozygosities based on the SNP chip were higher for all individuals outside Africa, but no different inside Africa populations. As noted above, this is expected as SNPs on the chip were chosen to equally represent Africa and outside Africa variants (Evans *et al*. 2015).

#### Population structure

The genetic distances we found between Africa and outside Africa populations are consistent with previously reported genetic distances for microsatellites (Gloria-Soria *et al*. 2016) and exome sequencing (Crawford *et al*. 2017). Although our between population Weir and Cockerham weighted *F*_ST_ estimates for the SNP chip are nearly double those of WGS, we found a significant, positive correlation between the 2 distances.

Despite the individual composition of the populations analyzed with each technology, we found similar population structure in the PCA and clustering analysis (Supplementary Figs. 3 and 4). Both SNP chip and WGS identified 2 main genetic clusters—Africa and outside Africa—and a third, intermediate cluster from Argentina. However, the SNP chip based PCA more clearly separated populations from Puerto Rico and México than that performed using WGS (Supplementary Fig. 3), reflecting the ability of the SNP chip to maximize differentiation among outside African populations.

#### Demographic analysis and phylogenetic signal

The same demographic scenario was suggested regardless of the genotyping method used. The ABC analysis suggests *A. aegypti* colonization from Africa to America occurred ca. 500 years ago with a subsequent invasion of Asian and Pacific Islands, a scenario congruent with those proposed by previous work (Evans *et al*. 2015; Gloria-Soria *et al*. 2016; Kotsakiozi, Evans, *et al*. 2018; Kotsakiozi, Gloria-Soria, *et al*. 2018; Evans *et al*. 2019; Soghigian *et al*. 2020). However, the WGS analyses exhibited considerably lower local errors and greater optimal scenario support than that of the SNP chip, indicating that WGS data may be more powerful for demographic analyses. We also observed that while the topology of phylogenies derived from both methods are identical, with 2 well-supported clades harboring Africa and outside Africa populations (Supplementary Fig. 6), secondary nodes were better supported in the WGS phylogeny. These differences are likely the consequence of differences in the SFS introduced during the SNP design, with rare allele frequency variants been abundant in the WGS dataset while the SNP chip is biased toward intermediate frequency alleles (Supplementary Fig. 2), as suggested by Lachance and Tishkoff (2013). Based on these results, we conclude that both methods could be used to investigate demographic history in this species, but when possible, WGS should be favored due to its increased discriminatory power (but also see: Crawford and Lazzaro 2012).

#### Correcting ascertainment bias

The Axiom aegypti1 SNP chip was designed by Evans *et al*. (2015) using 160 individuals from populations that represented the global distribution (i.e. Africa and outside Africa) of *A. aegypti*. Knowing that populations from Africa harbored greater genetic diversity, Evans *et al*. (2015) equalized SNP representation from 2 major clusters—36 specimens from Senegal and Uganda; 124 specimens from French Polynesia, Thailand, Australia, Mexico, Brazil, and USA—with the intention that it would be used on both African and outside Africa samples. Post hoc SNP chip design bias in the allele frequency spectrum can be corrected using numerical optimization and modeling the “true” or “unascertained” allele frequency distribution (Nielsen 2004; Nielsen *et al*. 2004; Kern 2009; Albrechtsen *et al*. 2010). However, this requires resequencing data and reliable information regarding the details of the ascertainment scheme (Nielsen 2004; Albrechtsen *et al*. 2010) which is not available for most of *A. aegypti* populations. Thus, we recommend researchers avoid using the SNP chip for any population inferences based on analyses of allele frequencies. Instead, we follow Lachance and Tishkoff (2013) and recommend its use for assignment-based analyses such as individual identification (i.e. genotype-based), genealogy (i.e. kinship-based), and genetic clustering approaches.

### Combining WGS and SNP chip derived genotypes

Overall, the high compatibility of the data generated by SNP chip and WGS genotyping in combined analyses suggests the ability to use SNP chip data in conjunction with WGS sequence data in the future to infer movement of populations. As sequencing costs decline, accessibility of WGS will continue to increase. By integrating WGS data with existing SNP chip samples, we were able to accurately assign WGS samples to population of origin for all populations except 1, Gabon. This is unsurprising, as recent results suggest that Gabon may in fact be a population admixed between east and west Africa genetic signatures (Rose *et al*. 2020). We predict that our assignment tests would be more sensitive if we had more WGS samples to work with.

### Computational and monetary resource considerations

An important consideration when deciding between SNP chip and WGS is sequencing cost, computational power, and data storage capacity. WGS sequencing cost has declined over the years and is expected to continue declining, although at the time the current study was done, costs per megabase have stagnated (Wetterstrand 2023) . The cost of SNP chip genotyping per individual is currently 70 USD, while WGS at 10× coverage for *A. aegypti* cost 182.72 USD—making the SNP chip a more affordable option. In addition, sequence data must be stored as media, and although storage costs are also dropping, they add an extra expense. The greater concern of data storage between SNP chip and WGS relates to the size of data files generated, which differ considerably between methods. Raw data generated from a SNP chip with 95 samples is approximately 360 MB (3.8 MB/individual) compared to an average of 4.3 GB per individual derived from short-read sequencing. Currently, our SNP chip dataset containing genotypes for approximately 3,200 individuals is about 600 MB, without the need of generating intermediate files. Meanwhile, for each individual sequenced by WGS, multiple, large intermediate files (e.g. BAM) must be produced prior to producing the final genotype file. These intermediate files often require 10s even 100s of GB of storage. Therefore, when computational resources are limited, the SNP chip has a considerable advantage relative to WGS and can address most of the research questions in population genetics.

### Conclusion and recommendation

The Axiom aegypti1 genotyping array possesses high reproducibly and can detect alternative alleles with higher sensitivity than a low-depth whole-genome sequencing approach. The ascertainment bias effect in allele frequency spectrum due to array construction tends to over-estimate parameters such as observed heterozygosity and fixation index such as *F*_ST_ in non-African *A. aegypti* populations. However, it produces congruent/scaled inter-population differentiation estimates when compared to a low-depth WGS approach. Moreover, the data generated from the SNP chip is compatible with WGS data in combined population genetic analyses. These results confirm that as sequencing costs decline and accessibility of WGS continues to increase, the databases can build on each other. We note that for the time being, the use of the Axiom aegypti1 genotyping array remains a reliable low-cost genotyping alternative to study *A. aegypti* populations.

## Supplementary Material

jkae082_Supplementary_Data

## Data Availability

Variant calling files (vcf) and PLINK variant files are available via Dryad (doi:10.5061/dryad.m0cfxppbd). Raw sequence reads have been deposited at NCBI Sequence Read Archive (SRA) BioProject PRJNA1081855. The scripts used to merge WGS and SNP chip data are provided in Supplementary File 1. Supplementary File 2 contains probe IDs of the SNPs from the chip that mapped uniquely to the chromosomes of the AaegL5 reference genome (Matthews *et al*. 2018) and the probe IDs of concordant SNPs between the SNP chip and low-coverage genome sequencing. Supplementary File 3 is a BED file with updated SNP chip coordinates based on the AaegL5 reference genome (Matthews *et al*. 2018). Supplemental material available at G3 online.
