# Supplementary material for: Robustness in population-structure and demographic-inference results derived from the Aedes aegypti genotyping chip and whole-genome sequencing data
Source: G3 (Bethesda). 2024 Apr 16;14(6):jkae082. doi: 10.1093/g3journal/jkae082 (PMC11152066; doi:10.1093/g3journal/jkae082)
Supplement: jkae082_Supplementary_Data [file jkae082_supplementary_data.zip › Figure_S2_G3-2024-404967.pdf]

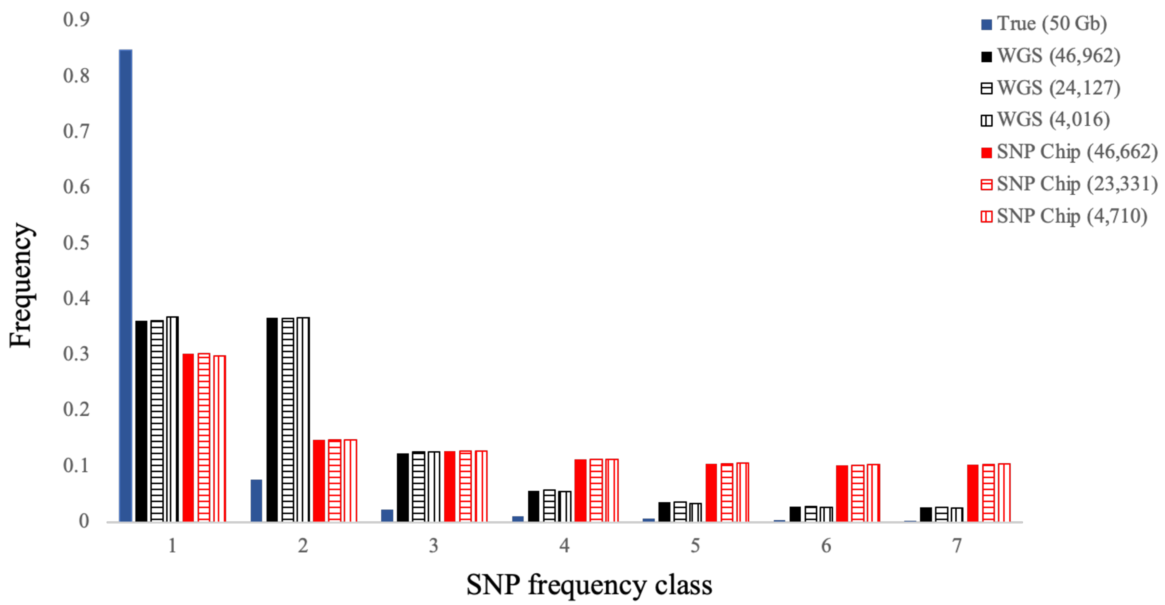

Figure S2 Site frequency spectrum for the corrected dataset, as well as for WGS and SNP chip-based subsets in 12 *Aedes aegypti* mosquitoes representing populations from Africa and outside Africa. Number of SNPs analyzed are indicated in parenthesis.
