# Supplementary material for: Robustness in population-structure and demographic-inference results derived from the Aedes aegypti genotyping chip and whole-genome sequencing data
Source: G3 (Bethesda). 2024 Apr 16;14(6):jkae082. doi: 10.1093/g3journal/jkae082 (PMC11152066; doi:10.1093/g3journal/jkae082)
Supplement: jkae082_Supplementary_Data [file jkae082_supplementary_data.zip › Figure_S3_G3-2024-404967.pdf]

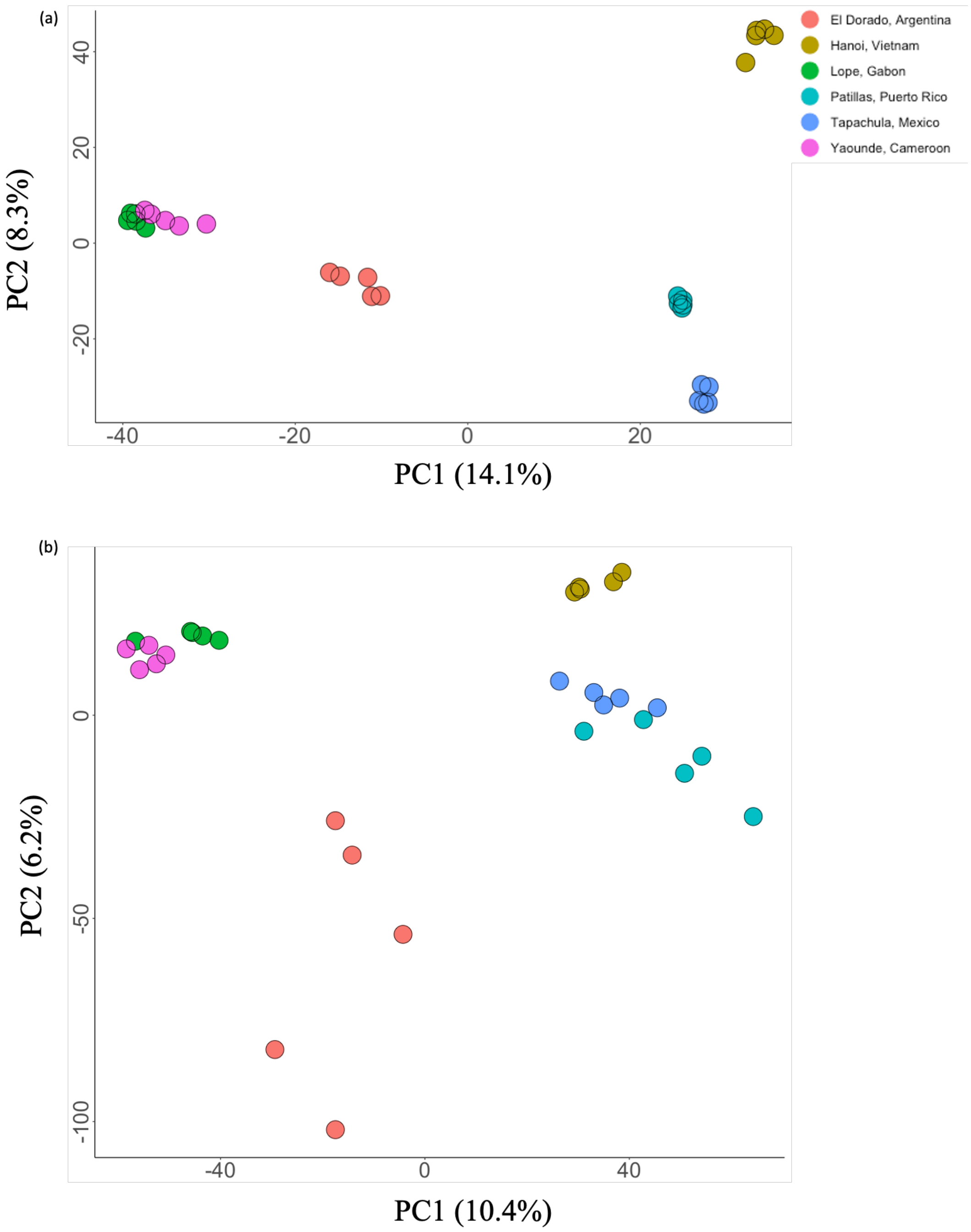

Figure S3. Principal Components Analysis (PCA) of *Aedes aegypti* populations. PCA was performed on 17 358 SNPs drawn from Axiom aegypti1 SNP chip (a) and (b) 178 004 SNPs drawn from low-depth whole genome sequencing approach. Individual mosquitoes are projected on the first two PCs. Populations are in different colors.
