# Supplementary material for: Robustness in population-structure and demographic-inference results derived from the Aedes aegypti genotyping chip and whole-genome sequencing data
Source: G3 (Bethesda). 2024 Apr 16;14(6):jkae082. doi: 10.1093/g3journal/jkae082 (PMC11152066; doi:10.1093/g3journal/jkae082)
Supplement: jkae082_Supplementary_Data [file jkae082_supplementary_data.zip › Figure_S5_G3-2024-404967.pdf]

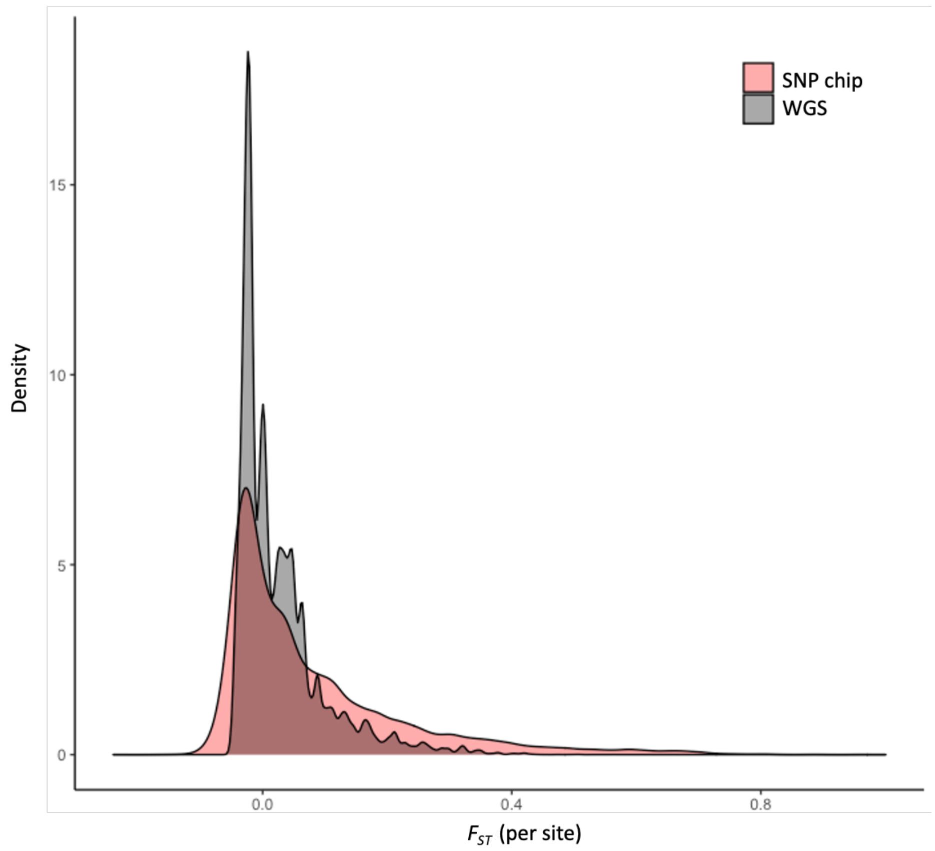

Figure S5 Weir and Cockerham F_{ST} per site estimated for all individuals from Africa and outside Africa groups according to Axiom aegypti1 SNP chip and low-depth whole genome sequencing approach as shown in the respective inset.
