# Supplementary material for: Robustness in population-structure and demographic-inference results derived from the Aedes aegypti genotyping chip and whole-genome sequencing data
Source: G3 (Bethesda). 2024 Apr 16;14(6):jkae082. doi: 10.1093/g3journal/jkae082 (PMC11152066; doi:10.1093/g3journal/jkae082)
Supplement: jkae082_Supplementary_Data [file jkae082_supplementary_data.zip › Figure_S7_G3-2024-404967.pdf]

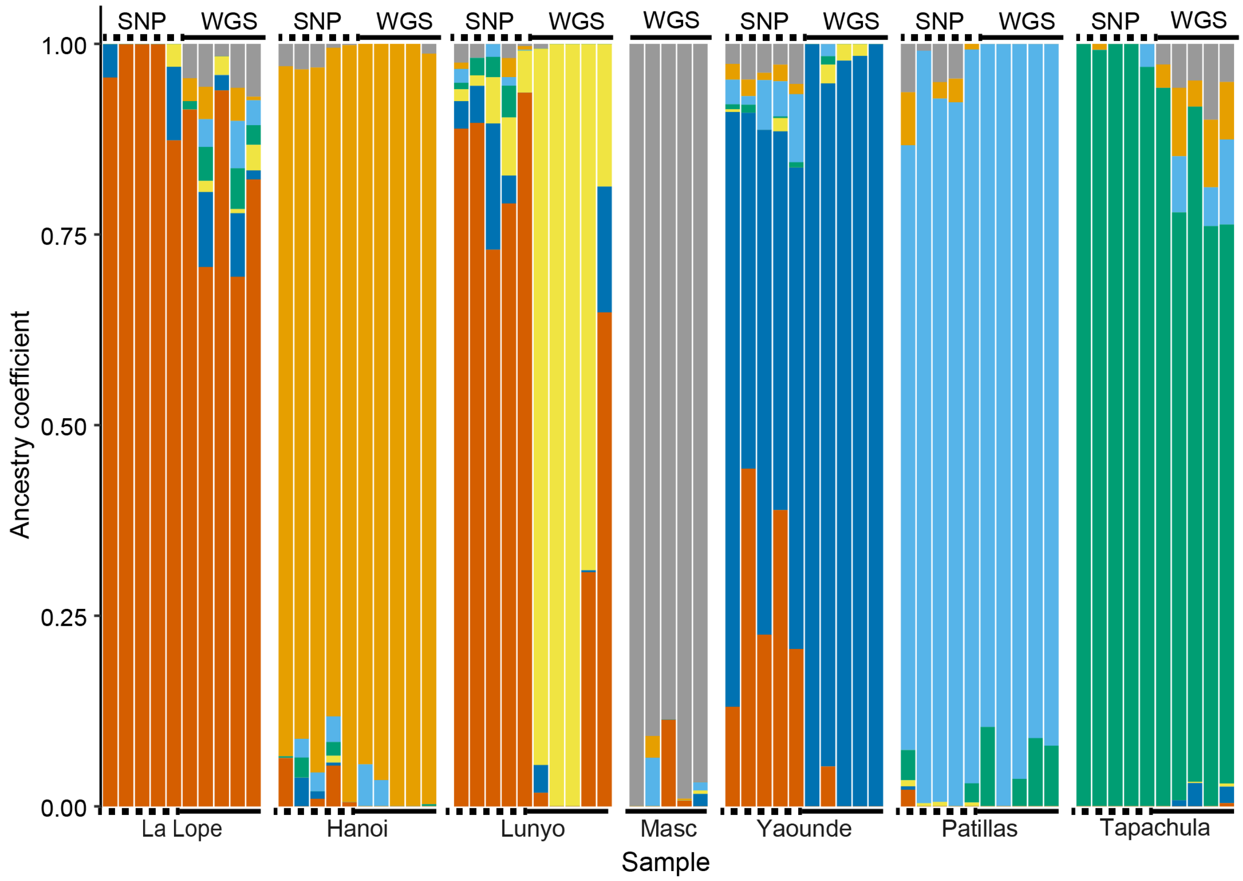

Figure S7. Ancestry coefficients for combined WGS and SNP chip dataset, for positions validated as concordant in this manuscript. Although K=4 was the best number of ancestral clusters based on cross entropy, we also present K=7 as this was the number of discreet populations used as an input to our SNMF analysis. Samples cluster by geographic region, regardless of genotyping method. Three letter population abbreviations: Masc – *Aedes mascarensis* outgroup (only WGS data), La Lope—La Lope, Gabon. Hanoi—Hanoi, Vietnam. Lunyo—Lunyo, Uganda. Yaounde —Yaounde, Cameroon. Patillas—Patillas, Puerto Rico. Tapachula—Tapachula Norte, Mexico.
