# Supplementary material for: Robustness in population-structure and demographic-inference results derived from the Aedes aegypti genotyping chip and whole-genome sequencing data
Source: G3 (Bethesda). 2024 Apr 16;14(6):jkae082. doi: 10.1093/g3journal/jkae082 (PMC11152066; doi:10.1093/g3journal/jkae082)
Supplement: jkae082_Supplementary_Data [file jkae082_supplementary_data.zip › Figure_S8_G3-2024-404967.pdf]

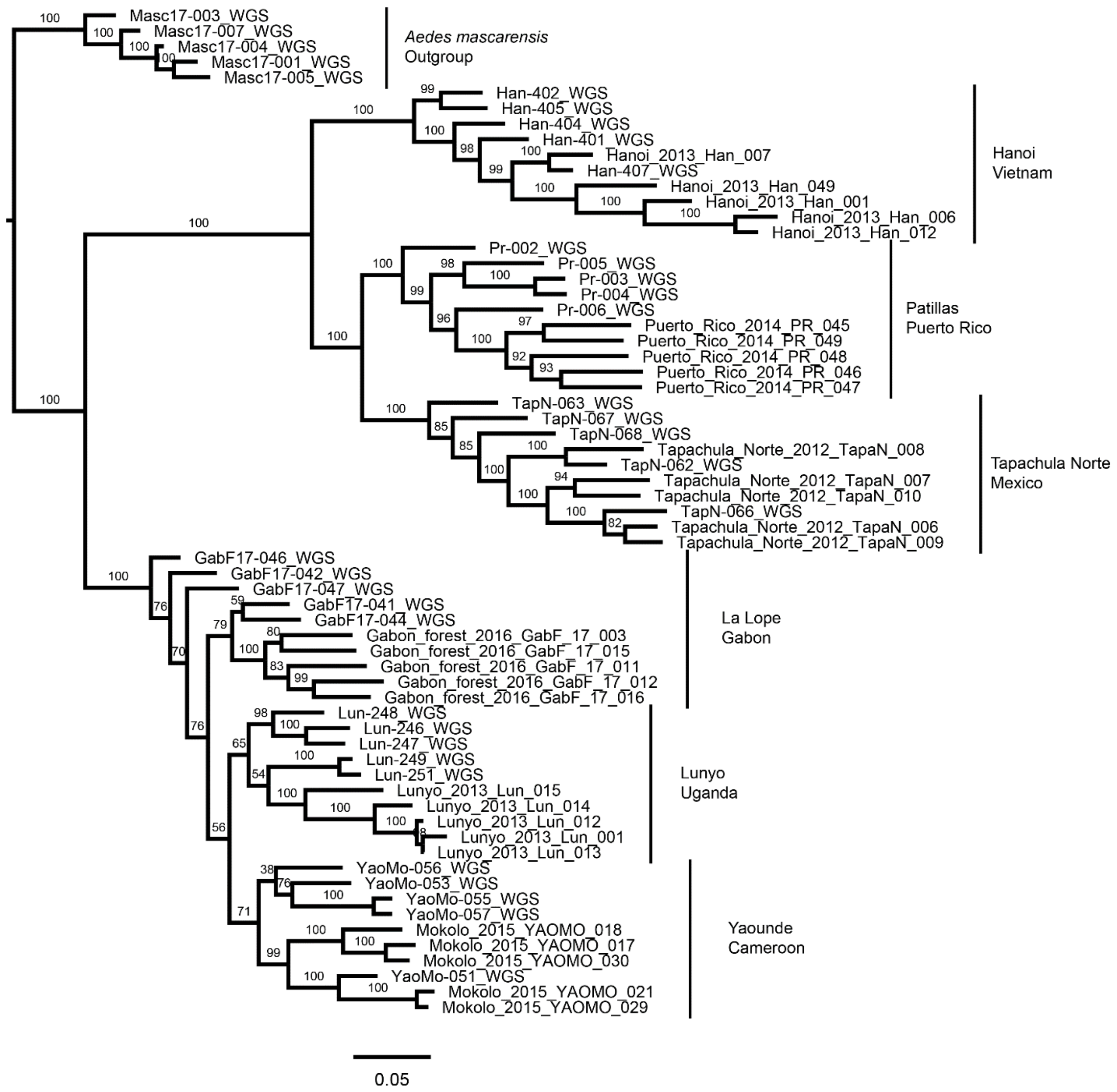

Figure S8 The maximum likelihood phylogeny inferred in IQ-Tree 2 based on the alignment of SNP sites validated as concordant in this manuscript. Samples cluster by population, regardless of genotyping method. Genotype data derived from the SNP chip is indicated by the suffix “SNP” after the sample identifier, while genotype data from whole genome sequencing is indicated by “WGS.” Support values are Ultrafast Bootstrap values. Branch lengths are in substitutions per site.
