# Supplementary material for: Robustness in population-structure and demographic-inference results derived from the Aedes aegypti genotyping chip and whole-genome sequencing data
Source: G3 (Bethesda). 2024 Apr 16;14(6):jkae082. doi: 10.1093/g3journal/jkae082 (PMC11152066; doi:10.1093/g3journal/jkae082)
Supplement: jkae082_Supplementary_Data [file jkae082_supplementary_data.zip › Figure_S9_G3-2024-404967.pdf]

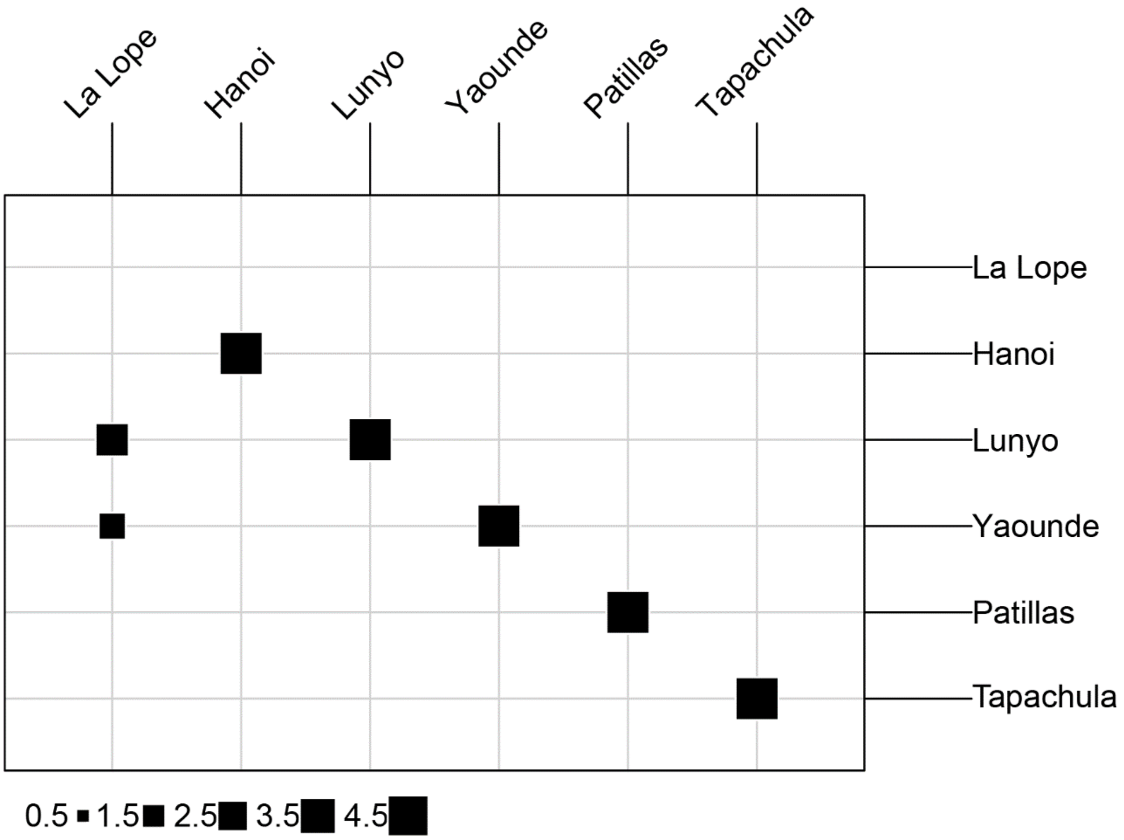

Figure S9 DAPC assignment tests for WGS samples with SNP samples as the training set, based on 11 PCA axes and 5 as the number of discriminant functions. All populations were accurately assigned (5/5 WGS samples), save for WGS samples from La Lope - Gabon, which were assigned to a mix of Lunyo and Yaounde populations. This is consistent with recent results suggesting Lope, Gabon may be an admixed population of east (represented by Lunyo, Uganda) and west (represented by Yaounde, Cameroon) African *Ae. aegypti* lineages (Rose et al. 2020). Populations: La Lope—La Lope, Gabon. Hanoi—Hanoi, Vietnam. Lunyo—Lunyo, Uganda. Yaounde —Yaounde, Cameroon. Patillas—Patillas, Puerto Rico. Tapachula—Tapachula Norte, Mexico.
