# Supplementary material for: Robustness in population-structure and demographic-inference results derived from the Aedes aegypti genotyping chip and whole-genome sequencing data
Source: G3 (Bethesda). 2024 Apr 16;14(6):jkae082. doi: 10.1093/g3journal/jkae082 (PMC11152066; doi:10.1093/g3journal/jkae082)
Supplement: jkae082_Supplementary_Data [file jkae082_supplementary_data.zip › Table_S10_G3-2024-404967.pdf]

Table S10 Pairwise genetic differentiation (*F_{ST}*) estimated for the dataset obtained from merging and filtering SNPs derived from the SNP chip and WGS data. Gab: La Lope – Gabon, Han: Hanoi - Vietnam, Lun: Lunyo - Uganda, Yao: Younde – Cameroon, PR: Patillas – Puerto Rico, Tap: Tapachula – Mexico, Mas: *Aedes mascarensis* (outgroup).

Population 1	Population 2	Fst
Gab_snp	Han_snp	0.410
Gab_snp	Lun_snp	0.138
Gab_snp	Yao_snp	0.109
Gab_snp	PR_snp	0.339
Gab_snp	Gab_wgs	0.035
Gab_snp	Han_wgs	0.385
Gab_snp	Lun_wgs	0.089
Gab_snp	Mas_wgs	0.303
Gab_snp	PR_wgs	0.342
Gab_snp	Tap_wgs	0.346
Gab_snp	Yao _wgs	0.104
Gab_snp	Tap_snp	0.367
Han_snp	Lun_snp	0.444
Han_snp	Yao_snp	0.388
Han_snp	PR_snp	0.175
Han_snp	Gab_wgs	0.402
Han_snp	Han_wgs	0.065
Han_snp	Lun_wgs	0.407
Han_snp	Mas_wgs	0.424
Han_snp	PR_wgs	0.184
Han_snp	Tap_wgs	0.208
Han_snp	Yao_wgs	0.365
Han_snp	Tap_snp	0.226
Lun_snp	Yao_snp	0.162
Lun_snp	PR _snp	0.378
Lun_snp	Gab_wgs	0.154
Lun_snp	Han_wgs	0.425
Lun_snp	Lun_wgs	0.145
Lun_snp	Mas_wgs	0.379
Lun_snp	PR_wgs	0.381
Lun_snp	Tap_wgs	0.388
Lun_snp	Yao_wgs	0.166
Lun_snp	Tap_snp	0.408
Yao _snp	PR_snp	0.317
Yao _snp	Gab_wgs	0.118
Yao _snp	Han_wgs	0.366

Yao _snp	Lun_wgs	0.126
Yao _snp	Mas_wgs	0.327
Yao _snp	PR_wgs	0.319
Yao _snp	Tap_wgs	0.327
Yao_snp	Yao_wgs	0.067
Yao_snp	Tap_snp	0.351
PR _snp	Gab_wgs	0.326
PR _snp	Han_wgs	0.175
PR _snp	Lun_wgs	0.328
PR _snp	Mas_wgs	0.358
PR _snp	PR_wgs	0.052
PR _snp	Tap_wgs	0.115
PR _snp	Yao _wgs	0.289
PR _snp	Tap_snp	0.112
Gab_wgs	Han_wgs	0.365
Gab_wgs	Lun_wgs	0.067
Gab_wgs	Mas_wgs	0.288
Gab_wgs	PR_wgs	0.316
Gab_wgs	Tap_wgs	0.319
Gab_wgs	Yao_wgs	0.072
Gab_wgs	Tap_snp	0.357
Han_wgs	Lun_wgs	0.374
Han_wgs	Mas_wgs	0.397
Han_wgs	PR_wgs	0.164
Han_wgs	Tap_wgs	0.180
Han_wgs	Yao_wgs	0.331
Han_wgs	Tap_snp	0.220
Lun_wgs	Mas_wgs	0.302
Lun_wgs	PR_wgs	0.331
Lun_wgs	Tap_wgs	0.333
Lun_wgs	Yao_wgs	0.078
Lun_wgs	Tap_snp	0.359
Mas_wgs	PR_wgs	0.357
Mas_wgs	Tap_wgs	0.364
Mas_wgs	Yao_wgs	0.288
Mas_wgs	Tap_snp	0.397
PR_wgs	Tap_wgs	0.103
PR_wgs	Yao_wgs	0.282
PR_wgs	Tap_snp	0.148
Tap_wgs	Yao_wgs	0.289
Tap_wgs	Tap_snp	0.027
Yao _wgs	Tap_snp	0.326
