# Supplementary material for: Robustness in population-structure and demographic-inference results derived from the Aedes aegypti genotyping chip and whole-genome sequencing data
Source: G3 (Bethesda). 2024 Apr 16;14(6):jkae082. doi: 10.1093/g3journal/jkae082 (PMC11152066; doi:10.1093/g3journal/jkae082)
Supplement: jkae082_Supplementary_Data [file jkae082_supplementary_data.zip › Table_S2_G3-2024-404967.pdf]

Table S2. Number of individuals analyzed with the Axiom aegypti1 SNP chip and whole genome sequencing methods. Note that individuals used for the population comparison between methods do not overlap. Furthermore, individuals from Mauritius are all *Aedes mascarensis* used as an outgroup and only from WGS.

				SNP chip		WGS	SNP chip & WGS	
Region	Country	Locale	Code	Genotype reproducibility	Population genomics	Population genomics	Concordance/Ascertainment analysis	Merged SNP chip & WGS
**Africa**	Angola	Luanda	Cu16				1*	
	Cameroon	Yaounde	YAOMO		5	5	1**	5
	Gabon	Lope	GabF_17		5	5		5
	France	Europa Island	EUR18				1	
	Kenya	Nairobi	Ken17				1	
	South Africa	Johannesburg	AFS				1	
	Sudan	Multiple loc.	SX20	10				
	Uganda	Lunyo	Lun		5[&]			5
	Mauritius	Port Louis	Masc17					5
**Asia**	Saudi Arabia	Jeddah	ASJ001				1	
	Sri Lanka	Multiple Loc.	FW, BW	10				
	Philippines	Cebu City	BBG				1	
	Vietnam	Hanoi	Han		5	5		5
**Europe**	Georgia	Tbilisi, Marneuli	GG				1	
**America**	Argentina	El Dorado	Dor		5	5		
		La Plata	LP				1	
		Posadas	Pos				1	
	France	Guadeloupe Island	Guad				1	
	Mexico	Tapachula	TapN		5	5	1	5
	United States	Patillas, Puerto Rico	PR		5	5		5
	United States	Tampa, Florida	Tam				1	

**Total**	20	30	30		12	35

* used only for the Concordance analysis

**used only for the Ascertainment bias analysis

[&] used only in the merge dataset. In grey to denote that is not included in the column total.
