# Supplementary material for: Robustness in population-structure and demographic-inference results derived from the Aedes aegypti genotyping chip and whole-genome sequencing data
Source: G3 (Bethesda). 2024 Apr 16;14(6):jkae082. doi: 10.1093/g3journal/jkae082 (PMC11152066; doi:10.1093/g3journal/jkae082)
Supplement: jkae082_Supplementary_Data [file jkae082_supplementary_data.zip › Table_S3_G3-2024-404967.pdf]

Table S3: Approximate Bayesian Computation analysis. Parameters used to identify the best colonization scenario using *Ae. aegypti* populations genotyped by SNP chip and WGS. Posterior estimations for effective population size and split times are shown only for the best supported scenario (Scenario 1 in bold).

Parameter	Description	Prior	Posterior	
			SNP chip	WGS
Colonization scenario	**Scenario 1: Africa to America to Asia**	**NA**	**0.636 (0.009)**	**0.851 (0.007)**
	Scenario 2: Africa to Asia to America	NA	0.155 (0.005)	0.076 (0.003)
	Scenario 3: Africa to America + Africa to Asia (after America colonization)	NA	0.042 (0.002)	0.029 (0.002)
	Scenario 4: Africa to America + Africa to Asia (before America colonization)	NA	0.130 (0.003)	0.082 (0.004)
Effective population size	Africa	100 - 500000	227824 (168081)	316435 (168861)
	America	100 - 500000	220366 (144193)	327120 (103556)
	Asia	100 - 500000	229540 (169180)	208611 (143020)
	Founding America hypothetical population (N2b)	10 - 1000	472 (244)	594 (251)
	Founding Asia hypothetical population (N3b)	10 - 1000	425 (287)	510 (256)
Split time - in generations (10 generations / year)	America from Africa	4500 - 6000	5282 (462)	5347 (419)
	Asia from America	1200 - 1800	1438 (168)	1526 (144)
