# Supplementary material for: Robustness in population-structure and demographic-inference results derived from the Aedes aegypti genotyping chip and whole-genome sequencing data
Source: G3 (Bethesda). 2024 Apr 16;14(6):jkae082. doi: 10.1093/g3journal/jkae082 (PMC11152066; doi:10.1093/g3journal/jkae082)
Supplement: jkae082_Supplementary_Data [file jkae082_supplementary_data.zip › Table_S4_G3-2024-404967.pdf]

Table S4 Reproducibility of SNP chip genotypes. After quality filtering 38,306 SNPs remained. Percentage (± SD%) of genotypes reproduced across three independent replicates of *Ae. aegypti* individuals genotyped using the Axiom aegypti1 SNP chip.

Country	Individual	Reproducibility
Sudan	SW20_010	93 (5)
	SW20_012	94 (4)
	SS20_010	96 (3)
	SS20_011	94 (5)
	SS20_012	95 (4)
	SD20_001	94 (4)
	SD20_003	94 (4)
	SD20_004	94 (4)
	SD20_008	94 (4)
	SD20_012	92 (6)
Sri Lanka	FW01_02	92 (6)
	FW01_03	95 (4)
	FW01_04	96 (3)
	FW03_03	95 (3)
	FW07_05	95 (4)
	BW01_01	95 (3)
	BW02_04	93 (5)
	BW02_05	95 (4)
	BW05_04	94 (4)
	BW09_02	91 (6)
Overall		94.5 (4.7)
