# Supplementary material for: Robustness in population-structure and demographic-inference results derived from the Aedes aegypti genotyping chip and whole-genome sequencing data
Source: G3 (Bethesda). 2024 Apr 16;14(6):jkae082. doi: 10.1093/g3journal/jkae082 (PMC11152066; doi:10.1093/g3journal/jkae082)
Supplement: jkae082_Supplementary_Data [file jkae082_supplementary_data.zip › Table_S5_G3-2024-404967.pdf]

Table S5. Genotype concordance between WGS and SNP chip summarized. 'Missing' is the difference between SNPs detected and the total number SNPs found on the chromosomes of the reference genome (43,953). HS (heterozygote sensitivity, shown as percentages) calculated as the proportion of concordant heterozygous sites found in one platform when the alternative allele was detected in the other.

Region	Locale	Sample	Avg. Depth	Avg. Quality Score	Concordant			SNPs Detected	Missing	HS_{SNP}	HS_{WGS}
					Het. (%)	Hom. (%)	Total (%)				
Africa	Luanda	Cu16002	5.60	105.50	2,393 (8.56)	25,577 (91.44)	27,970 (82.61)	33,858	9,735	84.56	34.33
	Europa Island	Europa5	6.61	121.51	917 (3.41)	25,967 (96.59)	26,884 (86.74)	30,992	12,601	80.44	22.69
	Nairobi	K17F46	6.81	119.92	2,219 (7.71)	26,545 (92.29)	28,764 (84.73)	33,947	9,646	82.03	36.48
	Johannesburg	AFS003	6.58	118.63	1,672 (5.81)	27,128 (94.19)	28,800 (86.51)	33,290	10,303	83.98	33.37
America	La Plata	LaPlata2	7.20	126.61	1,552 (5.97)	24,434 (94.03)	25,986 (74.09)	35,074	8,519	49.98	20.72
	Posadas	Pos001	7.74	129.73	3,016 (9.70)	28,090 (90.30)	31,106 (85.98)	36,180	7,413	85.2	44.56
	Guadeloupe Island	Guad1707	5.34	105.18	1,980 (7.34)	24,985 (92.66)	26,965 (81.78)	32,971	10,622	84.98	29.12
	Tapachula	TapN005	6.58	121.63	2,666 (8.42)	29,002 (91.58)	31,668 (86.31)	36,689	6,904	86.87	41.02
	Tampa	T7_25	5.77	108.00	2,562 (8.93)	26,130 (91.07)	28,692 (83.23)	34,474	9,119	86.5	36.21
Asia	Cebu City	BBG027	5.82	111.61	2,211 (7.32)	28,008 (92.68)	30,219 (85.92)	35,170	8,423	87.12	36.92
	Jeddah	AJS00110	6.12	116.46	1,995 (6.57)	28,373 (93.43)	30,368 (86.93)	34,932	8,661	86.97	36.51
Europe	Tbilisi	GG15	5.35	107.52	482 (2.14)	22,003 (97.86)	22,485 (67.79)	33,169	10,424	24.64	8.88
		Mean	6.30	116.03	1,972.08 (6.82)	26,353.5 (93.18)	28,325.58 (87.72)	34,228.83	9364.17	76.94	31.73
		SD	0.75	8.36	727.08 (2.28)	1,971.82 (2.28)	2,537.94 (5.79)	1,546.11	1546.11	19.39	9.93
