# Supplementary material for: Robustness in population-structure and demographic-inference results derived from the Aedes aegypti genotyping chip and whole-genome sequencing data
Source: G3 (Bethesda). 2024 Apr 16;14(6):jkae082. doi: 10.1093/g3journal/jkae082 (PMC11152066; doi:10.1093/g3journal/jkae082)
Supplement: jkae082_Supplementary_Data [file jkae082_supplementary_data.zip › Table_S6_G3-2024-404967.pdf]

Table S6. Generalized linear mixed model parameter odds-ratio estimates and model fit statistics. R^2_M and R^2_C are marginal and conditional R^2, which respectively account for variance explained by fixed effects alone and the total variance of the model. "Int" is the intercept, while "SD $Int._{Random}$" is the standard deviation of the random intercepts. For both models, *n* = 410,746 and df = 410,743.

Parameter	Estimate ± SE	Z	P	R^2_M	R^2_C	log(L)	AIC
Int.	5.193 ± 0.452	18.930	< 0.001	0.019	0.035	-181,885.390	363,776.775
Sequencing depth	1.456 ± 0.008	70.996	< 0.001				
SD $Int._{Random}$	0.340						
Int.	6.488 ± 0.634	19.148	< 0.001	0.113	0.130	-166,561.200	333,128.389
Quality score	2.59 ± 0.015	169.299	< 0.001				
SD $Int._{Random}$	0.370						
