# Supplementary material for: Robustness in population-structure and demographic-inference results derived from the Aedes aegypti genotyping chip and whole-genome sequencing data
Source: G3 (Bethesda). 2024 Apr 16;14(6):jkae082. doi: 10.1093/g3journal/jkae082 (PMC11152066; doi:10.1093/g3journal/jkae082)
Supplement: jkae082_Supplementary_Data [file jkae082_supplementary_data.zip › Table_S7_G3-2024-404967.pdf]

Table S7. Genome-based mapping parameters in 12 individuals of *Ae. aegypti* analyzed by the two methods.

Country	Locality	Code	Mapped reads (million)	Number SNPs	Mean depth (X)
Cameroon	Yaounde	YAOMO	35.3	208767	33.8
Europa Island	Europa	EUR18	32.0	208759	34.1
Kenya	Nairobi	Ken17	37.1	208780	33.0
South Africa	Johannesburg	AFS	35.9	208773	30.0
Saudi Arabia	Jeddah	ASJ001	36.4	208778	29.5
Philippines	Cebu City	BBG	36.0	208780	25.4
Georgia	Tbilisi, Marneuli	GG	33.5	208778	24.4
Argentina	La Plata	LP	38.4	208781	36.1
	Posadas	Pos	45.6	208781	38.2
France	Guadeloupe Island	Guad	32.7	208771	23.9
Mexico	Tapachula	TapN	40.8	208778	29.1
United States	Tampa	Tam	36.5	208778	27.7
Overall			36.7	208775	30.4
SD			3.5	6	4.5
