# Supplementary material for: Robustness in population-structure and demographic-inference results derived from the Aedes aegypti genotyping chip and whole-genome sequencing data
Source: G3 (Bethesda). 2024 Apr 16;14(6):jkae082. doi: 10.1093/g3journal/jkae082 (PMC11152066; doi:10.1093/g3journal/jkae082)
Supplement: jkae082_Supplementary_Data [file jkae082_supplementary_data.zip › Table_S8_G3-2024-404967.pdf]

Table S8. Mean heterozygosity estimated from subsets containing different numbers of SNPs drawn from 12 *Ae. aegypti* individuals analyzed by the two methods.

Region	Country	Locality	Code	Mean observed heterozygosity (Het)						
				Number of SNPs						
				Axiom aegypti1 chip			WGS			
				**46227**	**23331**	**4710**	**208775**	**46962**	**24127**	**4016**
Africa	Cameroon	Yaounde	YAOMO	0.180	0.184	0.183	0.181	0.173	0.171	0.171
	Europa Island	Europa	EUR18	0.161	0.161	0.159	0.185	0.172	0.170	0.161
	Kenya	Nairobi	Ken17	0.191	0.196	0.191	0.191	0.184	0.181	0.173
	South Africa	Johannesburg	AFS	0.164	0.165	0.167	0.178	0.170	0.170	0.169
	Saudi Arabia	Jeddah	ASJ001	0.163	0.164	0.161	0.150	0.143	0.142	0.134
Asia	Philippines	Cebu City	BBG	0.174	0.172	0.169	0.153	0.144	0.143	0.136
Europe	Georgia	Tbilisi, Marneuli	GG	0.171	0.169	0.176	0.149	0.140	0.140	0.138
America	Argentina	La Plata	LP	0.220	0.218	0.211	0.173	0.164	0.164	0.163
		Posadas	Pos	0.193	0.189	0.195	0.174	0.166	0.167	0.160
	France	Guadeloupe Island	Guad	0.216	0.218	0.218	0.156	0.150	0.150	0.146
	Mexico	Tapachula	TapN	0.183	0.184	0.183	0.158	0.150	0.150	0.152
	United States	Tampa	Tam	0.209	0.211	0.220	0.163	0.152	0.151	0.155
Overall				0.190	0.190	0.190	0.170	0.160	0.160	0.150
SD				0.020	0.020	0.020	0.010	0.010	0.010	0.010
