# Supplementary material for: Robustness in population-structure and demographic-inference results derived from the Aedes aegypti genotyping chip and whole-genome sequencing data
Source: G3 (Bethesda). 2024 Apr 16;14(6):jkae082. doi: 10.1093/g3journal/jkae082 (PMC11152066; doi:10.1093/g3journal/jkae082)
Supplement: jkae082_Supplementary_Data [file jkae082_supplementary_data.zip › Table_S9_G3-2024-404967.pdf]

Table S9 Weir and Cockerham pairwise F_{ST} matrix of six *Ae. aegypti* populations from Africa and outside Africa. Upper, shaded triangle of the matrix shows pairwise F_{ST} for WGS, while the lower triangle shows pairwise F_{ST} for the SNP chip.

	Yaounde, Cameroon	La Lope, Gabon,	El Dorado, Argentina	Hanoi, Vietnam	Patillas, Puerto Rico,	Tapachula, Mexico
Yaounde, Cameroon		0.043	0.065	0.119	0.102	0.105
La Lope, Gabon	0.082		0.074	0.127	0.110	0.109
El Dorado, Argentina	0.139	0.139		0.083	0.064	0.067
Hanoi, Vietnam	0.244	0.251	0.213		0.063	0.068
Patillas, Puerto Rico	0.185	0.194	0.144	0.150		0.042
Tapachula, Mexico	0.219	0.226	0.179	0.200	0.105	
