# Supplementary material for: Robustness in population-structure and demographic-inference results derived from the Aedes aegypti genotyping chip and whole-genome sequencing data
Source: G3 (Bethesda). 2024 Apr 16;14(6):jkae082. doi: 10.1093/g3journal/jkae082 (PMC11152066; doi:10.1093/g3journal/jkae082)
Supplement: jkae082_Supplementary_Data [file Figure_S6_G3-2024-404967.pdf › Figure_S6_G3-2024-404967.pdf]

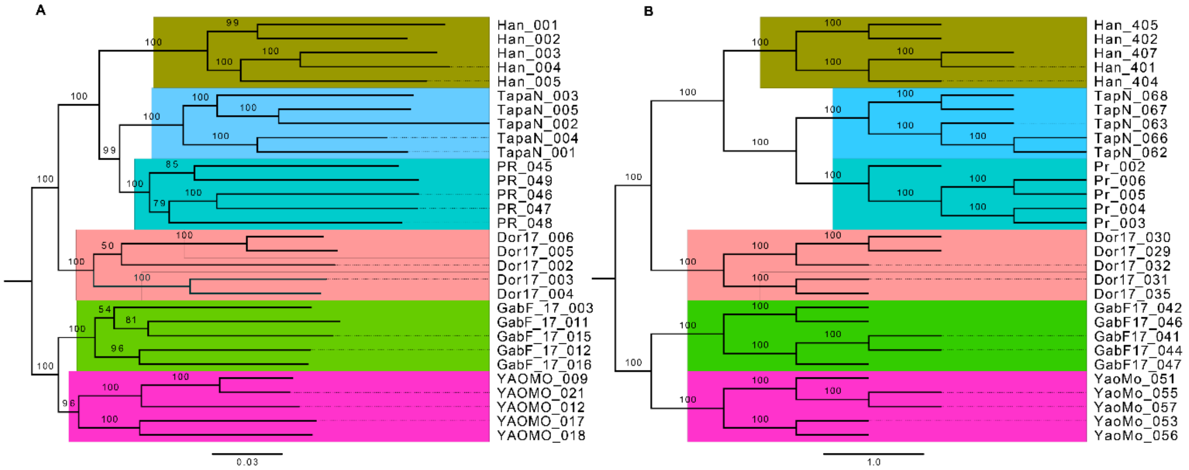

Figure S6. Maximum-likelihood consensus phylogenetic tree of *Ae. aegypti* mosquitoes from Africa and outside Africa populations built using (A) 17,358 SNPs from Axiom aegypti1 chip and (B) 178,004 SNPs from low-depth whole genome sequencing. Colors highlight individuals from the same population. Numbers on branches are bootstrap values. Population codes as in Table S1.
